# Evaluation of *In Vitro* Anti-Inflammatory Activities and Protective Effect of Fermented Preparations of Rhizoma Atractylodis Macrocephalae on Intestinal Barrier Function against Lipopolysaccharide Insult

**DOI:** 10.1155/2013/363076

**Published:** 2013-03-14

**Authors:** Shambhunath Bose, Hojun Kim

**Affiliations:** ^1^College of Oriental Medicine, Dongguk University, Gyeongju, Republic of Korea; ^2^College of Pharmacy, Dongguk University, Goyang, Seoul 410-820, Republic of Korea; ^3^Department of Oriental Rehabilitation Medicine, Dongguk University, Graduate School of Oriental Medicine, 814 Siksa-dong, Goyang, Gyeonggi-do Seoul 410-773, Republic of Korea

## Abstract

Lipopolysaccharide (LPS), a potent inducer of systemic inflammatory responses, is known to cause impairment of intestinal barrier function. Here, we evaluated the *in vitro* protective effect of an unfermented formulation of Rhizoma Atractylodis Macrocephalae (RAM), a traditional Chinese herbal medicine widely used in the treatment of many digestive and gastrointestinal disorders, and two fermented preparations of RAM, designated as FRAM-1 (prepared in Luria-Bertani broth) and FRAM-2 (prepared in glucose), on intestinal epithelial cells (IECs) against LPS insult. In general, fermented formulations, especially FRAM-2, but not unfermented RAM, exerted an appreciable protective effect on IECs against LPS-induced perturbation of membrane resistance and permeability. Both fermented formulations exhibited appreciable anti-inflammatory activities in terms of their ability to inhibit LPS-induced gene expression and induced production of a number of key inflammatory mediators and cytokines in RAW 264.7 macrophage cells. However, in most cases, FRAM-2 exhibited stronger anti-inflammatory effects than FRAM-1. Our findings also suggest that suppression of nuclear factor-**κ** **β** (NF-**κ** **β**) activity might be one of the possible mechanisms by which the fermented RAM exerts its anti-inflammatory effects. Collectively, our results highlight the benefits of using fermented products of RAM to protect against LPS-induced inflammatory insult and impairment in intestinal barrier function.

## 1. Introduction

The intestinal epithelium, which is composed of a single layer of cells, functions as a selectively permeable barrier, allowing the absorption of nutrients, electrolytes, and water while preventing passage of larger, potentially toxic, compounds and/or enteric flora from the lumen [1]. Disruption of intestinal epithelial permeability, resulting in development of leaky gut, has been implicated in the pathogenesis of several gastrointestinal diseases, including food allergies, inflammatory bowel disease (IBD), and irritable bowel syndrome (IBS) [2]. The toxins produced by enteric pathogens are among the most potent agents causing damage to intestinal permeability [1]. It has been found that LPS or endotoxin, an integral component of the outer membrane of Gram-negative bacteria and one of the most abundant proinflammatory stimuli on the gastrointestinal tract, could cause impairment of intestinal barrier function [3]. This event may, in turn, result in augmentation of intestinal permeability, facilitating bacterial translocation from the gut lumen to mesenteric lymph nodes or other organs [4–6]. Eventually, this process leads to the development of systemic sepsis and multiple-organ dysfunction syndrome (MODS) [7]. 

Accumulating evidence suggests the direct interaction of LPS with IECs via Toll-like receptor [8]. LPS is known to enhance cellular oxidative stress [9] via the generation of reactive oxygen species (ROS) [10], which could impair barrier function through disruption of epithelial tight junctions (TJs) [11, 12] and induction of epithelial cell apoptosis [13]. LPS also induces the intestinal expression and enzymatic activity of both cyclooxygenase-2 (COX-2) and inducible nitric oxide synthase (iNOS) [14, 15], which generate free radicals, such as reactive oxygen species (ROS) and nitric oxide (NO) [16]. NO can disrupt the intestinal barrier through a number of mechanisms, including membrane peroxidation and apoptosis of cells [3].

Additionally, LPS is also known to induce several inflammatory responses [17–19] which can be mediated via induction of oxidative stress. This event may lead to tissue damage and increased epithelial permeability [20]. Free radicals and reactive oxygen metabolites trigger and/or amplify inflammation via the upregulation of expression of a number of genes, including NF-*κβ* [21, 22]. Activation of NF-*κβ* in turn leads to amplification of the inflammatory response by upregulating production of several proinflammatory cytokines and enzymes, such as interleukin- (IL-) 1, IL-6, tumor necrosis factor alpha (TNF-*α*), and iNOS [21, 22].

Taking the above information into consideration, it is conceivable that scavenging of free radicals by appropriate antioxidants might be a useful approach to combating endotoxin-mediated disruption of intestinal barrier function. Although synthetic antioxidants are widely used, their safety and toxicity issues are a major concern. Therefore, much attention has been focused on the use of natural antioxidants. A number of studies have shown that many plant and herbal extracts and their products, such as polyphenolic substances (e.g., flavonoids and tannins), exert potent antioxidant actions. RAM, an herb utilized in various dietary preparations in Asian countries, has been reported to possess antioxidant activities [23, 24]. It has been shown that RAM prevents viral gastroenteritis via the protection of intestinal mucosal cells against injury and improvement in the absorptive function [25]. 

Fermentation generally causes breakdown or conversion of undesirable substrates into compatible components, thereby improving product properties via increasing the level of many bioactive compounds. The beneficial health effects of probiotics and their fermented food products are well known [26]. The fermentation process has been shown to improve the antioxidant properties of plants and vegetables [27, 28]. More specifically, fermentation increases the phenolic content of plant products [28] and a positive correlation between the polyphenols and antioxidant activities of herbs has been reported [29]. In parallel, fermentation can also augment the anti-inflammatory activities of food as well as plant and herbal products [30–34]. In a recent study, we demonstrated that fermentation significantly improved the protective effects of RAM, either alone *in vitro* or in combination with other herbs *in vivo* against LPS insult [18]. 

The above findings prompted us to evaluate the question of whether the permeability of IECs under LPS insult could be modulated by RAM upon fermentation. In order to address this issue from the mechanistic point of view, we attempted to determine whether fermentation could improve the antioxidant activity of RAM. To elucidate further, we also evaluated the impact of FRAMs on gene expression and production of key inflammatory mediators and enzymatic activities of COX-2 and iNOS using the RAW264.7 murine macrophage cell line, which is a widely used *in vitro* model for the study of inflammatory responses. Based on our earlier findings [18], the fermentation of RAM was performed using *Bacillus licheniformis*, which is listed in the third edition of the Food Chemicals Codex (1981) as a source of carbohydrase and protease enzymes. This bacterial species has been used safely for large-scale industrial fermentation as well as in commercial human and animal probiotic products [35, 36]. In addition, as the type of fermentation can determine the degree of modification and the level of the most bioactive compounds in plant products [28], the fermentation of RAM was performed in either Luria-Bertani (LB) broth or in glucose solution in order to determine whether the fermentation conditions could influence the protective effects of FRAM against LPS insult.

## 2. Methods and Materials

### 2.1. Herbal Extraction and Fermentation

Dried RAM was procured from the Department of Medicine of Dongguk International Hospital (Goyang, Republic of Korea). The extraction and fermentation of this herb were performed according to our laboratory-optimized procedure, as described previously [18]. Briefly, 20 g of the herb was mixed with 200 mL of boiled Milli-Q water, and this mixture was ultrasonicated at 70°C to disperse the particles and then incubated at 70°C for 3 h in a water bath under continuous shaking. Subsequently, the samples dedicated for fermentation were supplemented with either LB broth (2.5% w/v, for FRAM-1) or glucose (2% w/v, for FRAM-2), and the resultant mixtures were vigorously vortexed. The products were then autoclaved for 20 min at 121°C in order to sterilize the samples and to facilitate decoction of the samples. After cooling the preparations to room temperature, the samples were inoculated with a fresh subculture (2% v/v) of *B. licheniformis *and fermented for 24 h at 31°C. The unfermented extract (RAM) was prepared in a similar way, except that it was not supplemented with LB broth or glucose and was not subjected to *B. licheniformis*-mediated fermentation. The unfermented and fermented preparations were then subjected to low speed centrifugation in order to sediment the particles. The resultant supernatants were sterilized by filtration through a 0.2 *μ*m pore filter (Sartorius, USA) and were stored in aliquots at −20°C until used in the experiments.

### 2.2. Determination of Free Radical Scavenging Activity of the Herbal Preparations by 1,1-Diphenyl-2-picrylhydrazyl (DPPH) Radical Scavenging Assay

The free radical scavenging activity of the herbal formulations was determined using the stable free radical DPPH. Briefly, a 2.5 *μ*L aliquot of each herbal preparation, which was diluted to 100 *μ*L, was added to 100 *μ*L of 60 *μ*M DPPH solution (prepared in ethanol) in a 96-well microtiter plate and mixed thoroughly. The reaction mixture was incubated in the dark for 30 min at room temperature. The absorbance of the wells was then measured at 540 nm on a microplate reader (Spectramax Plus, Molecular Devices, Sunnyvale, CA, USA). The radical scavenging activity of the samples was expressed as % inhibition of DPPH absorbance using the following equation:

(1) Inhibition(%)  =[1−(Asample  −  Asample  blank)Acontrol]×100,

where *A*_control_ was the absorbance of the control (DPPH solution without test sample), *A*_sample_ was the absorbance of the test sample (DPPH solution plus test sample), and *A*_sample  blank_ was the absorbance of the sample only (sample without DPPH solution).

### 2.3. Determination of Total Polyphenol Content of the Herbal Preparations

Total polyphenolic content of the herbal formulations was assessed using the Folin-Denis colorimetric method [37] with some modification. Briefly, 10 *μ*L of each herbal sample was added to 790 *μ*L of water in microcentrifuge tubes and mixed thoroughly. To this, 50 *μ*L of Folin-Denis reagent (Sigma-Aldrich, St. Louis, MO, USA) was added, followed by vigorous mixing. One minute later, 150 *μ*L of 20% sodium carbonate solution was added and the contents were mixed thoroughly. The reaction mixture was then incubated in the dark for 1 h at room temperature; the tubes were then centrifuged for 5 min at 3000 rpm. An aliquot of the resultant supernatant was transferred to the individual well of a 96-well microtiter plate and the absorbance was read at 750 nm using a microplate reader (Spectramax Plus). A calibration curve was prepared using gallic acid (Sigma-Aldrich) as a standard, which was used further for determination of total phenolics in the samples. The data were expressed as mg gallic acid equivalent (GAE) per g of the extracted herb.

### 2.4. Cell Culture

The murine macrophage RAW264.7 cell line (American Type Culture Collection, ATCC, Rockville, MD, USA) was maintained in Dulbecco's Modified Eagle Medium (DMEM) supplemented with 10% heat inactivated fetal bovine serum, 2 mM L-glutamine, 100 U/mL penicillin, and 100 *μ*g/mL streptomycin. The human colorectal carcinoma HCT-116 cell line (ATCC) was grown in McCoy's 5A medium (modified, Invitrogen Carlsbad, CA, USA) containing HEPES and L-glutamine and supplemented with 10% heat inactivated fetal bovine serum, 100 U/mL penicillin, and 100 *μ*g/mL streptomycin. Both cell lines were cultured in an incubator at 37°C under a humidified atmosphere of air containing 5% CO_2_. 

### 2.5. Measurement of TEER of HCT-116 Cells

Epithelial integrity of HCT-116 cells grown as monolayers on Millicell-24 cell culture insert plates (inserts: 12 mm in diameter, 0.4 *μ*m membrane pore size; Millipore, Bedford, MA, USA) was evaluated by the measurement of TEER using a Millicell ERS-2 epithelial volt-ohm meter (Millipore) and a STX01 chopstick-style electrode (Millipore). The cells were seeded onto the apical wells of the insert plates (2 × 10^5^ cells per well) filled with 400 *μ*L growth medium (HCT-116 culture medium, as described above), while the basolateral wells of the inserts were filled with 800 *μ*L of growth medium. The cells were allowed to grow in the inserts at 37°C under a humidified atmosphere containing 5% CO_2_ until they formed a confluent monolayer. The cells were then exposed for 24 h to the individual herbal formulation at concentrations equivalent to 50, 100, and 200 *μ*L of the extracted herbal preparations per mL of the cell culture medium. The control cells (N) and the cells that were assigned to treatment with LPS alone (LPS control) were exposed to sterile saline instead of the herbal extracts. After these treatments, LPS (from *Pseudomonas aeruginosa*, Sigma-Aldrich), which was prepared in sterile PBS at pH 7.4, was added to the wells at a final concentration of 10 *μ*g/mL, except for the control (N) wells (which received PBS alone). The cells were incubated in this condition for additional 24 h, followed by performance of TEER measurement, according to the instructions of the manufacturer of the instrument (Millipore). The electrical resistance of the monolayers was measured by the electrode by placing its shorter tip in the plate insert and the longer tip in the outer well. The resistance of inserts without cells (blank resistance) was subtracted from that of the experimental inserts in order to obtain the actual electrical resistance of the epithelial cell monolayer. TEER values were calculated according to the following equation: TEER = resistance × filter area (*Ω* × cm^2^).

### 2.6. Measurement of HRP Flux in HCT-116 Cells

This assay was performed in order to determine the effect of LPS alone or in combination with the herbal formulations on the permeability of LPS-treated HCT-116 cells. For this study, only a 200 *μ*L/mL concentration of the herbal preparation was chosen because, at this concentration, all herbal preparations exhibited their maximum protective effect against LPS-induced change in membrane resistance (see Section 3). The cells were seeded onto the apical wells of Millicell-24 cell culture insert plates (Millipore) at a density of 2 × 10^5^ cells per well and grown until they formed a confluent monolayer, as described above. After attaining confluence, the cells were treated for 24 h with the individual herbal formulation at a concentration of 200 *μ*L/mL. The control cells (N) and the cells that were assigned to treatment with LPS alone (LPS control) were exposed to sterile saline instead of the herbal formulations. Following these treatments, LPS (from *Pseudomonas aeruginosa*, Sigma-Aldrich) in sterile PBS (pH 7.4) was added to the wells at a final concentration of 10 *μ*g/mL, except for the control (N) wells (treated with PBS alone). The cells were incubated in this condition for an additional 24 h; the cells were then washed with Hanks Balanced Salt Solution (HBSS) without phenol red (Invitrogen), and, finally, both apical and basolateral compartments of the inserts were filled with HBSS without phenol red. HRP (Sigma-Aldrich) was then added to the apical wells at a final concentration of 0.15 mg/mL. The cells were incubated in this condition for 1 h, followed by collection of 10 *μ*L of solution from the basolateral compartment. The HRP content of the samples was determined spectrophotometrically (Spectramax Plus) in a 96-well microtiter plate by assaying peroxidase activity using 3,3′,5,5′-tetramethylbenzidine (TMB; Sigma-Aldrich) as an HRP substrate. The HRP flux of the noncontrol samples was expressed as % of that of control.

### 2.7. Cytotoxicity Assessment of RAW264.7 Cells

The cytotoxicity of FRAMs for RAW264.7 cells was assessed by a colorimetric assay using 3-(4,5-dimethylthiazol-2-yl)-2,5 diphenyltetrazolium bromide (MTT) as the chromophore. Following three to four cycles of subculturing, the cells were seeded into 24-well plates at a density of 2 × 10^5^ cells/well. The cells were incubated in this condition overnight, followed by treatment with FRAM formulations at concentrations equivalent to 5, 10, 25, 50, and 100 *μ*L of the extracted herbal preparations per mL of cell culture medium for 24 h. The control cells (N) were exposed to sterile saline instead of the fermented herbal extract. Three hours prior to the end of the treatment schedule, MTT was added to the cells at a final concentration of 0.5 mg/mL. Following completion of the MTT reaction, the culture media were carefully removed from the wells, and DMSO was added to the cells in order to release and dissolve the formazan crystal products. Following this reaction, the absorbance was read at 570 nm using a microplate reader (Spectramax Plus). The viability of the control cells, in terms of their absorbance, was expressed as 100%.

### 2.8. Determination of Expression of COX-2, iNOS, TNF-*α*, IL-1*β*, and IL-6 Genes in RAW264.7 Cells by Quantitative Real-Time PCR (qRT-PCR)

Following three to four cycles of subculturing, RAW264.7 cells were seeded at a density of 8 × 10^5^ cells/well in 6-well plates. After growing overnight, the cells were treated with FRAM formulations at concentrations equivalent to 50 and 100 *μ*L of the extracted herbal preparations per mL of cell culture medium for 24 h. The control cells (N) and the cells that were assigned to treatment with LPS alone (LPS control, LC) were exposed to sterile saline instead of the FRAM formulations. LPS (from *Pseudomonas aeruginosa*, Sigma-Aldrich), which was prepared in sterile PBS at pH 7.4, was then added to the wells at a final concentration of 10 *μ*g/mL, except for the control (N) wells (received PBS alone). The cells were then incubated for additional 24 h prior to their use in the gene expression experiments.

Total RNA was extracted from the cells using a commercial Trizol reagent kit (Invitrogen) according to the kit manufacturer's instructions. The purity and concentration of the extracted RNA were determined using spectrophotometry. For generation of cDNA, an equal quantity of each RNA preparation (2 *μ*g) was reverse transcribed using a Sprint RT Complete Oligo-(dT)_18_ cDNA synthesis kit (Clontech, Mountain View, CA, USA) according to the instructions provided by the kit manufacturer. qRT-PCR of the samples was performed in a LightCycler instrument (Roche Applied Science, Indianapolis, ID, USA) using a LightCycler FastStart DNA Master SYBR Green kit (Roche Applied Science). The amplification reactions were performed in accordance with the manufacturer's instructions, in a total reaction volume of 20 *μ*L containing the PCR mix, 1 *μ*L of cDNA, and gene-specific primers (10 pmol for each). The sequences of the primers (Bioneer, Daejeon, Korea) used in our experiment were as follows—COX-2-forward: 5′-AGAAGGAAATGGCTGCAGAA-3′ and COX-2-reverse: 5′-GCTCGGCTTCCAGTATTGAG-3′ [38]; iNOS-forward: 5′-AGCCCAACAATACAAGATGACCCTA-3′ and iNOS-reverse: 5′-TTCCTGTTGTTTCTATTTCCTTTGT-3′ [39]. TNF-*α*-forward: 5′-GAACTGGCAGAAGAGGCACT-3′ and TNF-*α*-reverse: 5′-AGGGTCTGGGCCATAGAACT-3′ [40]; IL-1*β* forward: 5′-GCCCATCCTCTGTGACTCAT-3′ and IL-1*β* reverse: 5′-AGGCCACAGGTATTTTGTCG-3′ [41]; IL-6 forward: 5′-AGTTGCCTTCTTGGGACTGA-3′ and IL-6 reverse: 5′-CAGAATTGCCATTGCACAAC-3′ [42]; glyceraldehyde 3-phosphate dehydrogenase (GAPDH)-forward: 5′-TGATGACATCAAGAAGGTGGTGAAG-3′ and GAPDH-reverse: 5′-TCCTTGGAGGCCATGTAGGCCAT-3′ [43]. The annealing temperatures of the primers for the PCR reactions that were optimized prior to the assay were 53°C (for COX-2), 56°C (for GAPDH and iNOS), and 60°C (for TNF-*α*, IL-1*β*, and IL-6). The following conditions were used for the PCR amplification reactions: an initial incubation step at 95°C for 10 min, followed by 30 amplification cycles, each one consisting of a denaturation step at 95°C for 10 s (COX-2, TNF-*α*, IL-1*β*, and IL-6) or 30 s (GAPDH and iNOS), an annealing step at the corresponding optimized annealing temperature for 10 s (COX-2, TNF-*α*, IL-1*β*, and IL-6) or 30 s (GAPDH and iNOS), and an extension step at 72°C for 15 s (COX-2, TNF-*α*, IL-1*β*, and IL-6) or 90 s (GAPDH and iNOS). Following this reaction, a melting curve analysis was performed in order to verify the specificity of the amplicon. The LightCycler software supplied by the instrument manufacturer (Roche Applied Science) was used for processing and analyzing the data. The relative expression of genes was quantitated following the standard 2^−Δ*c*_*t*_^ calculation using the housekeeping gene, GAPDH, for normalization, where *C*_*t*_ denotes the crossing threshold value calculated by the software and Δ*C*_*t*_ = (*C*_*t*-target gene_ − *C*_*t*-GAPDH_).

### 2.9. Measurement of Production of NO, PGE_2_, TNF-*α*, IL-1*β*, and IL-6 by RAW 264.7 Cells

For all of the following assays, the samples consisted of the media collected from the culture of cells that were used for analysis of expression of previously described inflammatory genes. Production of NO, in terms of nitrite secretion by the cells, was measured colorimetrically using Griess reagent (Promega, Madison, WI, USA). Briefly, after termination of the desired treatments, 100 *μ*L of culture medium from each well of the plate was mixed with an equal volume of Griess reagent followed by incubation at room temperature for 10 min. The absorbance was read at 540 nm using a microplate reader (Spectramax Plus), and the nitrite concentration of each sample was determined using a freshly prepared sodium nitrite standard curve. Prostaglandin E2 (PGE_2_) was determined by a colorimetric assay using a PGE_2_ assay kit (R&D Systems, Minneapolis, MN, USA), in accordance with the kit manufacturer's instructions. TNF-*α* was measured by ELISA using a mouse-specific TNF-*α* ELISA Kit (Komabiotech, Seoul, Republic of Korea), and IL-1*β* and IL-6 were determined by colorimetric assays using mouse-specific immunoassay kits (R&D Systems) according to the manufacturer's instructions.

### 2.10. NF-*κβ* Activation Assay

In our study, FRAM-2 showed stronger *in vitro* anti-inflammatory activities than FRAM-1 (see Section 3). To further elucidate the mode of action of FRAM-2 in the inflammatory signaling cascade, we studied the impact of this formulation on the LPS-induced NF-*κβ* activity of the cells. Briefly, after three to four cycles of subculturing, RAW264.7 cells were seeded at a density of 1 × 10^6^ cells/well in 6-well plates. Following overnight growth, the cells were treated for 24 h with FRAM-2 at concentrations equivalent to 50 and 100 *μ*L of the extracted herbal preparation per mL of cell culture medium. The cells serving as control (N) and the cells that were assigned to treatment with LPS alone (LC) were exposed to sterile saline instead of the fermented herbal extract. After this treatment, LPS (used in the above-mentioned gene expression study) was added to the wells at a final concentration of 10 *μ*g/mL, except for the control (N) wells (which received PBS alone). The cells were then incubated for 1 h and were then harvested for isolation of nuclear protein fractions, as described previously [44], with some modifications. Briefly, after collection, the cells were dispersed in a hypotonic buffer (10 mM HEPES, pH 7.9; 10 mM KCl; 1 mM DTT; 2 mM MgCl_2_; 1 mM PMSF; 20 *μ*L/mL protease inhibitor cocktail (Sigma-Aldrich); 0.1% TritonX-100) for 15 min on ice, followed by vigorous vortexing for 10 s. The preparations were centrifuged at 15,000 g for 10 min at 4°C to pellet the nuclei. The nuclei were subsequently resuspended in a hypertonic buffer (20 mM HEPES, pH 7.9; 25% glycerol; 420 mM NaCl; 2 mM MgCl_2_; 1 mM DTT; 0.2 mM ETDA; 1 mM PMSF; 20 *μ*L/mL protease inhibitor cocktail) and incubated on ice for 30 min. The isolated nuclear fractions were centrifuged at 15,000 g for 10 min at 4°C, and the resultant supernatants containing nuclear proteins were collected. The prepared nuclear extracts were then used to detect the DNA binding activity of NF-*κβ* using a NF-*κβ* (p65) Transcription Factor Assay Kit (Cayman Chemical Company, Ann Arbor, MI, USA) according to the manufacturer's protocol.

### 2.11. Statistical Analyses

The values are expressed as the mean ± SD. The statistical package for social science (SPSS) software program (version 17.0; SPSS, Chicago, IL, USA) was used for analysis of data. One-way ANOVA followed by Bonferroni's post hoc test was performed in order to determine significant differences between the study groups. Post hoc analyses were performed only when the means were significantly different by one-way ANOVA. When the error variance was found to be heterogeneous using Levene's test, a logarithmic transformation of the raw data was performed and is indicated accordingly in Section 3. The differences were considered significant when *P* < 0.05.

## 3. Results 

### 3.1. Impact of the Herbal Formulations on TEER and Membrane Permeability of LPS-Treated HCT-116 Cells

**Figure 1:**
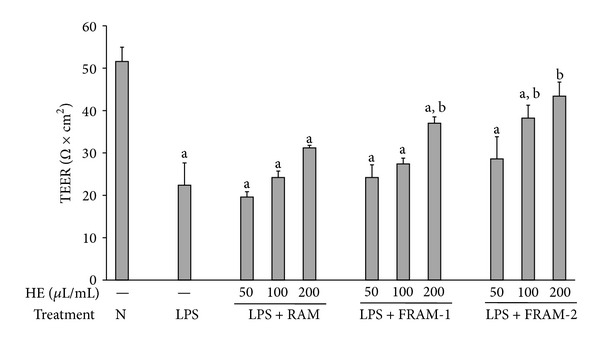
The impact of treatment with saline or with different concentrations of unfermented Rhizoma Atractylodis Macrocephalae (RAM) or fermented RAM-1 (FRAM-1) and fermented RAM-2 (FRAM-2) formulations on the transepithelial electrical resistance (TEER) of HCT-116 cells exposed to LPS. The cells were treated with the indicated concentrations of RAM and FRAMs for 24 h. The control cells (N) and the noncontrol cells that were assigned to treatment with LPS alone (LPS) were treated with sterile saline instead of herbal extracts. After this treatment, the noncontrol and control cells were treated with LPS (10 *μ*g/mL) and PBS, respectively, for 24 h, followed by the performance of TEER measurement. The detailed treatment regimen and experimental conditions are described in Section 2. The data are expressed as the mean ± SD, *n* = 3. ^a^Statistically significant difference compared to control cells (*P* < 0.05); ^b^statistically significant difference compared to cells treated with LPS plus saline (*P* < 0.05). HE: herbal extract.

**Figure 2:**
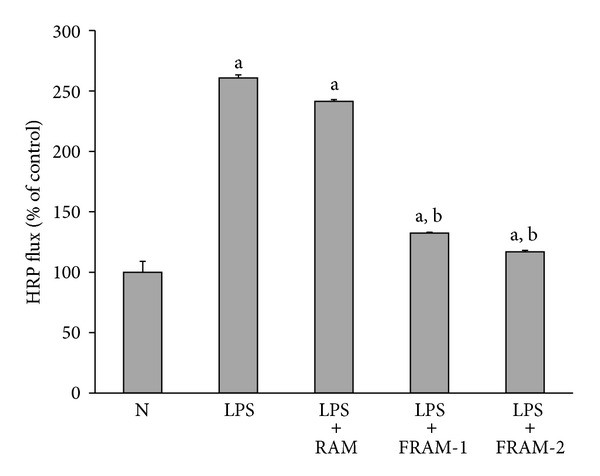
The impact of treatment with saline or with different concentrations of unfermented Rhizoma Atractylodis Macrocephalae (RAM) or fermented RAM-1 (FRAM-1) and fermented RAM-2 (FRAM-2) formulations on the HRP-flux of HCT-116 cells exposed to LPS. The cells were treated with RAM and FRAMs at a concentration of 200 *μ*L/mL for 24 h. The control cells (N) and the noncontrol cells that were assigned to treatment with LPS alone were treated with sterile saline instead of herbal extracts. After this treatment, the noncontrol and control cells were treated with LPS (10 *μ*g/mL) and PBS, respectively, for 24 h, followed by HRP-flux measurement. The detailed treatment regimen and experimental conditions are described in Section 2. The data are expressed as the mean ± SD, *n* = 3. The data were log-transformed prior to analysis by ANOVA. ^a^Statistically significant difference compared to control cells (*P* < 0.05); ^b^statistically significant difference compared to cells treated with LPS plus saline (*P* < 0.05).

Treatment of cells with LPS resulted in significant changes (*P* < 0.05) in TEER (57% decline, Figure 1) and membrane permeability in terms of HRP flux (161% increase, Figure 2). Although a decline of 13% and an increase of 8% and 39% in TEER were observed in LPS-treated cells in response to coexposure to RAM at 50, 100, and 200 *μ*L/mL concentrations, respectively, these changes were found to be statistically insignificant (*P* > 0.05). TEER was augmented in LPS-treated cells by being treated with both fermented RAMs in a concentration-dependent manner. However, these increments were significant at a 200 *μ*L/mL concentration of FRAM-1 (65% increase) and at 100 and 200 *μ*L/mL concentrations of FRAM-2 (71% and 94% enhancement, resp.). In assessment of the effect of the herbal formulations on the transcellular flux of HRP using a 200 *μ*L/mL concentration of the herb, the permeability of LPS-treated cells was decreased insignificantly by treatment with RAM (7% reduction, *P* > 0.05) but significantly by treatment with both FRAM-1 (49% decrease, *P* < 0.05) and FRAM-2 (55% depletion, *P* < 0.05).

### 3.2. Antioxidant Activity of the Herbal Formulations

**Figure 3:**
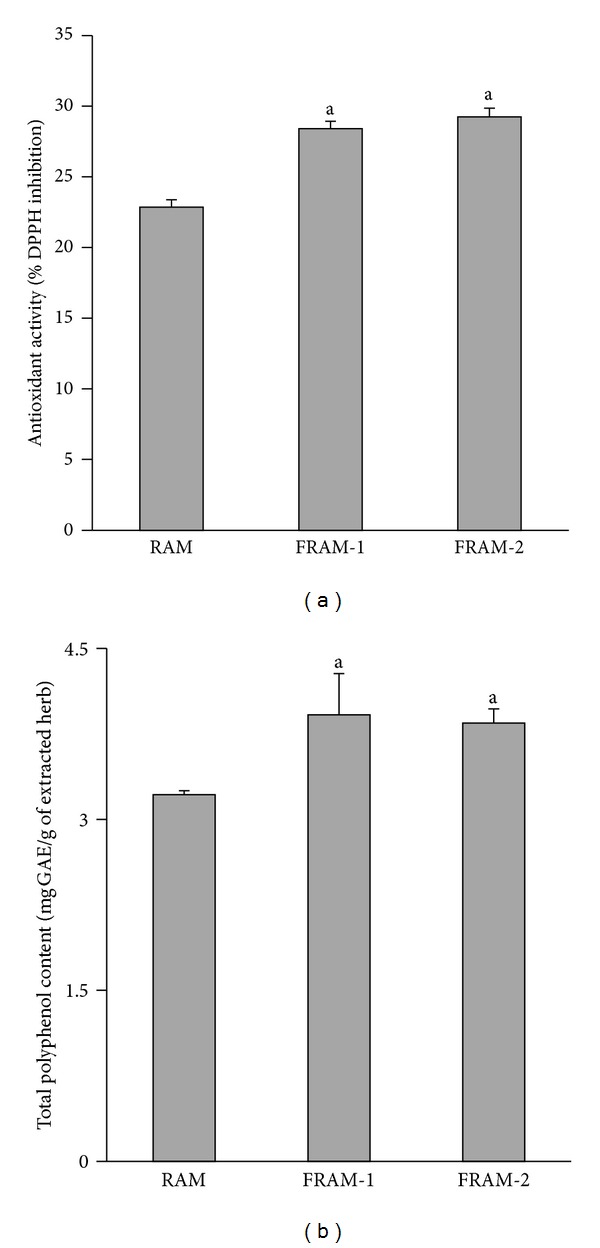
The DPPH radical scavenging activity (a) and total polyphenol content (b) of unfermented Rhizoma Atractylodis Macrocephalae (RAM) or fermented RAM-1 (FRAM-1) and fermented RAM-2 (FRAM-2) formulations. The detailed treatment regimen and experimental conditions are described in Section 2. The data are expressed as the mean ± SD, *n* = 3. ^a^Statistically significant difference compared to RAM (*P* < 0.05).

RAM exhibited approximately 23% inhibition of DPPH radical formation (Figure 3(a)), while both FRAMs showed significantly higher DPPH radical scavenging activity compared to RAM (*P* < 0.05). In keeping with this, the total polyphenol content of RAM, which was estimated to be 3.2 mg GAE per g of the extracted herb, was also significantly augmented (*P* < 0.05) because of prior fermentation, without being influenced by the conditions of fermentation (Figure 3(b)). 

### 3.3. Anti-Inflammatory Activities of the Fermented Herbal Formulation: Impact on Gene Expression and Production of Key Inflammatory Mediators and Production of NO and PGE_2_

**Figure 4:**
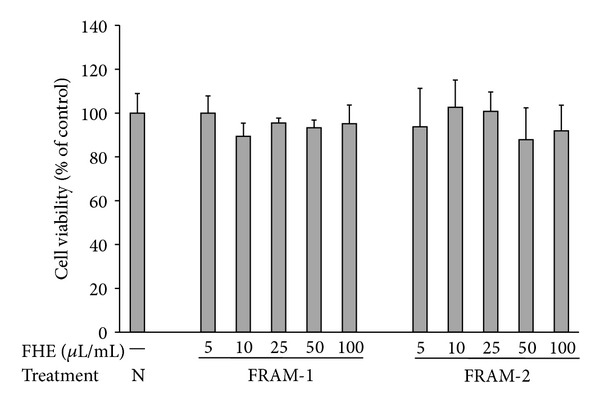
The impact of treatment with saline (N) or with different concentrations of fermented Rhizoma Atractylodis Macrocephalae-1 (FRAM-1) or fermented Rhizoma Atractylodis Macrocephalae-2 (FRAM-2) formulations on the viability of RAW264.7 cells. The cells were treated for 24 h with the indicated concentrations of FRAMs followed by the assessment of cell viability. The detailed treatment regimen and experimental conditions are described in Section 2. The viability of the cells that were treated with saline was set to 100%. The data are expressed as the mean ± SD, *n* = 4. No statistically significant differences were observed between the treatment groups. FHE: fermented herbal extract.

Because both FRAM-1 and FRAM-2 exerted stronger protection of IECs against LPS insult and exhibited greater antioxidant activity and polyphenol content, compared to RAM, we next evaluated the anti-inflammatory activity of the two above-mentioned FRAMs using a RAW264.7 cell line as a model and LPS as the inflammation-inducing agent. A concentration-dependent cytotoxicity assessment was performed for selection of the optimal nonlethal concentrations of the FRAMs to be used in this study. Accordingly, in comparison with the control, treatment with either FRAM formulation did not result in a significant change in the viability of the cells (Figure 4). This finding was valid at concentrations as high as 100 *μ*L of the fermented herbal preparations per mL of the cell culture medium. Subsequently, 50 and 100 *μ*L/mL concentrations of the FRAMs were used for evaluation of their anti-inflammatory activities. 

**Figure 5:**
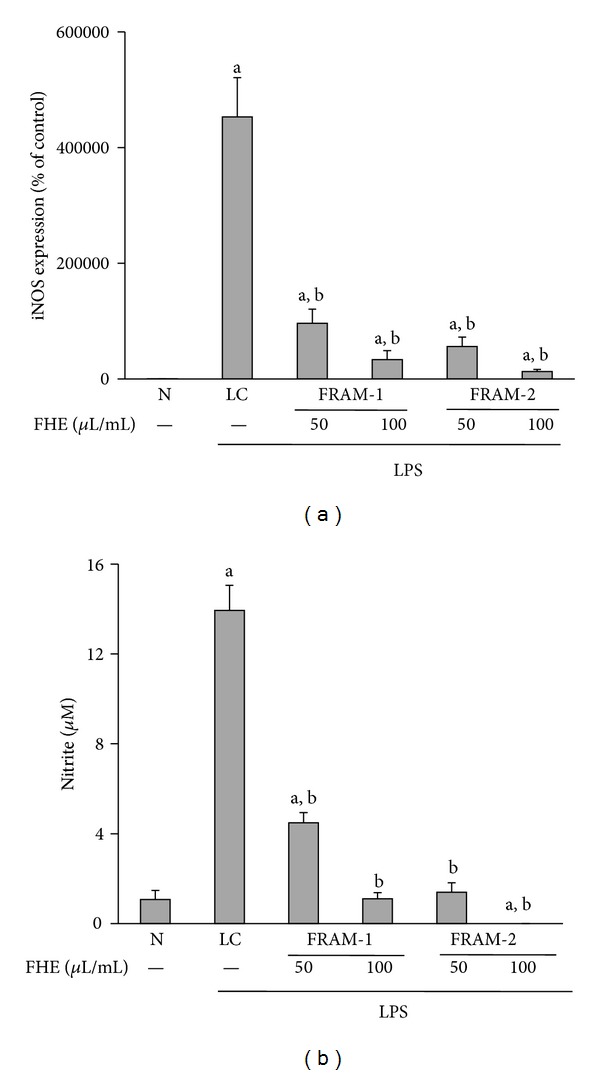
The effect of treatment with LPS in combination with saline (LC) or with two different concentrations of fermented Rhizoma Atractylodis Macrocephalae-1 (FRAM-1) and fermented Rhizoma Atractylodis Macrocephalae-2 (FRAM-2) formulations on the expression of the iNOS gene (a) and production of nitrite (b) in RAW264.7 cells. The cells were treated for 24 h with the indicated concentrations of FRAMs. The control cells (N) and the noncontrol cells that were assigned to treatment with LPS alone (LC) were treated with sterile saline instead of herbal extracts. After this treatment, the noncontrol and control cells were treated with LPS (10 *μ*g/mL) and PBS, respectively, for 24 h, followed by the determination of iNOS gene expression and nitrite production. The detailed treatment regimen and experimental conditions are described in Section 2. The level of iNOS gene expression in control cells (N) was set to 100%. The data are expressed as the mean ± SD, *n* = 3. The data for both iNOS and nitrite production were log-transformed prior to analysis by ANOVA. ^a^Statistically significant difference compared to control cells (*P* < 0.05); ^b^statistically significant difference compared to cells treated with LPS plus saline (*P* < 0.05). FHE: fermented herbal extract.

Exposure of cells to LPS consistently provoked a marked increase in both iNOS transcription (4531-fold, Figure 5(a)) and NO production (13 fold, Figure 5(b)) (*P* < 0.05 for both). The two parameters described above were significantly inhibited in LPS-treated cells (*P* < 0.05) when the cells were cotreated with either of the FRAM formulations at both experimental concentrations. However, maximal inhibition of these LPS-induced parameters was observed following treatment with FRAM-2. Exposure to the lower and higher concentrations of this formulation led to inhibition of LPS-induced transcription of iNOS by 88% and 97%, respectively, and LPS-induced production of NO by 89% and below the limit of detection, respectively. On the other hand, coexposure of LPS-treated cells to the lower and higher concentrations of FRAM-1 resulted in inhibition of iNOS transcription by 79% and 93%, respectively, and NO production by 68% and 92%, respectively.

**Figure 6:**
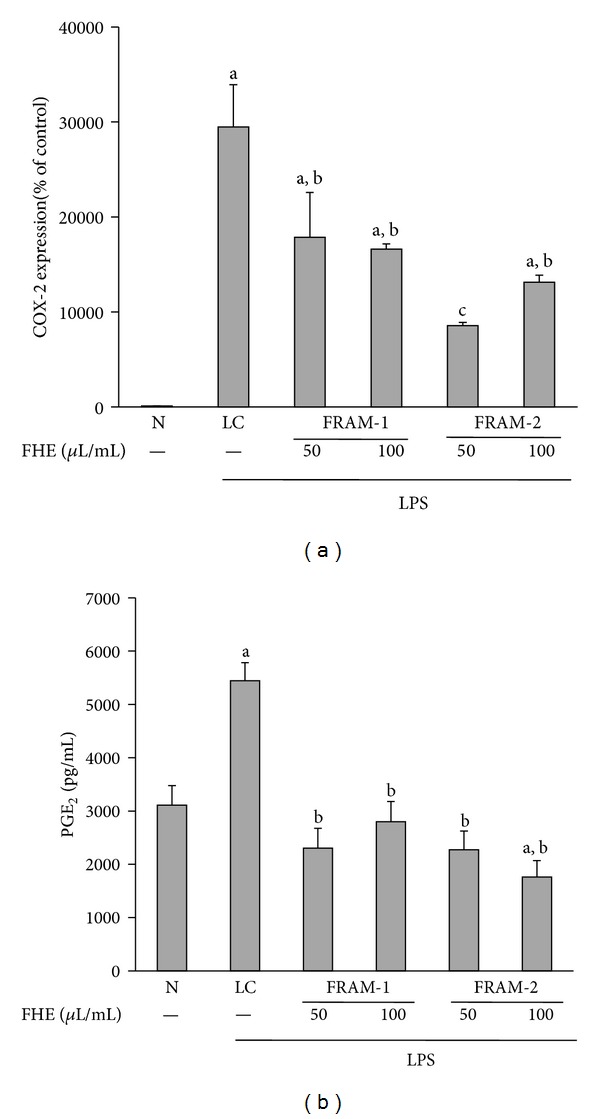
The effect of treatment with LPS in combination with saline (LC) or with two different concentrations of fermented Rhizoma Atractylodis Macrocephalae-1 (FRAM-1) and fermented Rhizoma Atractylodis Macrocephalae-2 (FRAM-2) formulations on the expression of the COX-2 gene (a) and production of PGE_2_ (b) in RAW264.7 cells. The cells were treated with the indicated concentrations of FRAMs for 24 h. The control cells (N) and the noncontrol cells that were assigned to treatment with LPS alone (LC) were treated with sterile saline instead of herbal extracts. After this treatment, the noncontrol and control cells were treated with LPS (10 *μ*g/mL) and PBS, respectively, for 24 h, followed by determination of COX-2 gene expression and PGE_2_ production. The detailed treatment regimen and experimental conditions are described in Section 2. The level of COX-2 gene expression in the control cells (N) was set to 100%. The data are expressed as the mean ± SD, *n* = 3. The data for COX-2 expression were log-transformed prior to analysis by ANOVA. ^a^Statistically significant difference compared to control cells (*P* < 0.05); ^b^statistically significant difference compared to cells treated with LPS plus saline (*P* < 0.05); ^c^significantly lower compared to all other treatment groups (*P* < 0.05). FHE: fermented herbal extract.

Treatment of cells with LPS led to a dramatic increase in COX-2 transcription (294 fold enhancement, Figure 6(a)), as well as moderate augmentation in PGE_2_ production (75% increase, Figure 6(b)) (*P* < 0.05 for both). Treatment with each FRAM preparation resulted in significant attenuation (*P* < 0.05) of LPS-induced transcription of COX-2 and production of PGE_2_ at both lower and higher concentrations. However, between the two formulations, the lower concentration of FRAM-2 caused maximum inhibition (71%) of LPS-induced transcription of COX-2. The level of COX-2 transcription did not differ significantly (*P* > 0.05) among LPS-treated cells coexposed to the lower and higher concentrations of FRAM-1 (40% and 44% inhibition, resp.) and the higher concentration of FRAM-2 (55% inhibition). On the other hand, LPS-induced production of PGE_2_ did not vary significantly among or between the LPS + FRAM-1 (58% and 49% inhibition by the lower and higher concentrations, resp.) and LPS + FRAM-2 treatments (58% and 68% inhibition by the lower and higher concentrations, resp.). 

**Figure 7:**
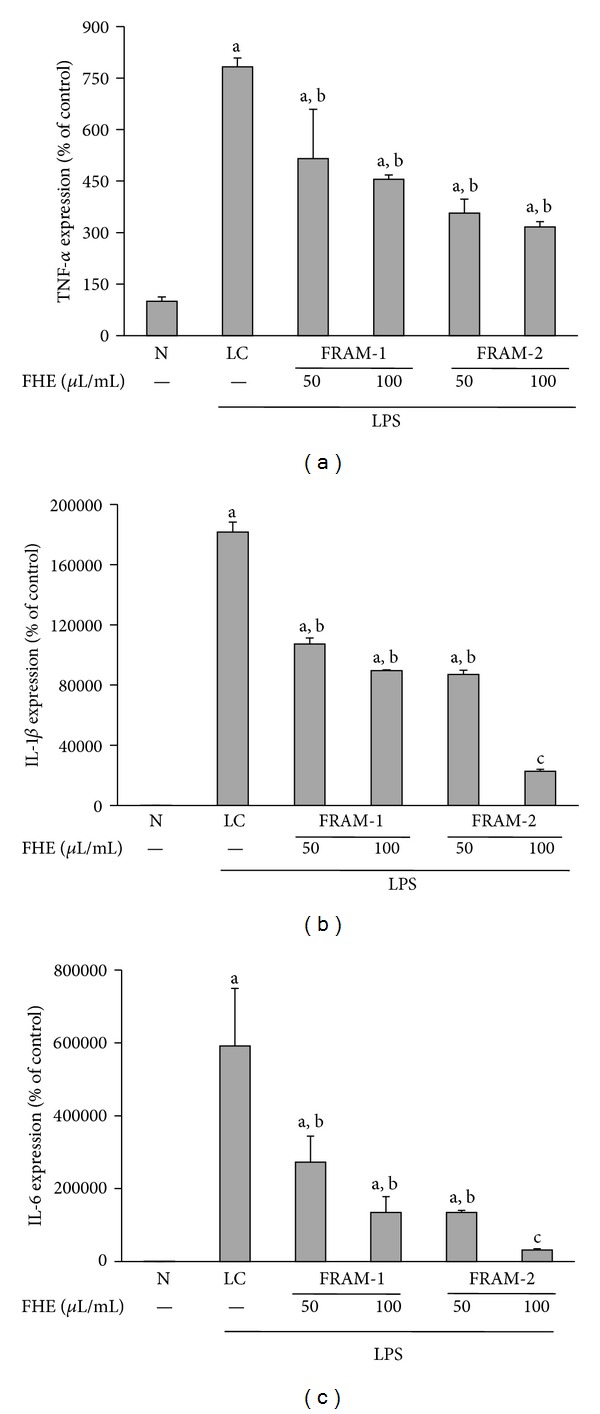
The effect of treatment with LPS in combination with saline (LC) or with two different concentrations of fermented Rhizoma Atractylodis Macrocephalae-1 (FRAM-1) and fermented Rhizoma Atractylodis Macrocephalae-2 (FRAM-2) formulations on expression of TNF-*α* (a), IL-1*β* (b), and IL-6 (c) genes in RAW264.7 cells. The cells were treated with the indicated concentrations of FRAMs for 24 h. The control cells (N) and the noncontrol cells that were assigned to treatment with LPS alone (LC) were treated with sterile saline instead of herbal extracts. After this treatment, the noncontrol and control cells were treated with LPS (10 *μ*g/mL) and PBS, respectively, for 24 h, followed by determination of gene expression of the above-mentioned cytokines. The detailed treatment regimen and experimental conditions are described in Section 2. The values are expressed as the mean ± SD, *n* = 3. The data for expression of all genes were log-transformed prior to analysis by ANOVA. ^a^Statistically significant difference compared to control cells (*P* < 0.05); ^b^statistically significant difference compared to cells treated with LPS plus saline (*P* < 0.05); ^c^significantly lower compared to all other treatment groups (*P* < 0.05). FHE: fermented herbal extract.

Treatment of cells with LPS resulted in a marked increase in expression of the TNF-*α* gene (683% enhancement, Figure 7(a)) (*P* < 0.05). However, cotreatment of cells with both fermented herbal formulations resulted in significantly decreased LPS-induced transcription of TNF-*α* (*P* < 0.05). More specifically, expression of TNF-*α* in LPS-treated cells was inhibited by 34% and 42% by the lower and higher concentrations of FRAM-1, respectively, and by 54% and 60% by the lower and higher concentrations of FRAM-2, respectively.

Treatment with LPS resulted in a dramatic increase in IL-1*β* transcription in the cells (1817 fold, Figure 7(b)) (*P* < 0.05). However, co-treatment of the cells with both fermented herbal formulations resulted in significant attenuation of LPS-induced expression of the IL-1*β* gene (*P* < 0.05), accounting for 41% and 51% inhibition by the lower and higher concentrations of FRAM-1, respectively, and 52% and 88% inhibition by the lower and higher concentrations of FRAM-2, respectively. Notably, the level of IL-1*β* transcription in LPS + FRAM-2-treated cells at the higher concentration of FRAM-2 was significantly lower (*P* < 0.05) compared to all other treatment groups. 

Exposure of cells to LPS also resulted in a remarkable increase in expression of the IL-6 gene (5920-fold, Figure 7(c)) (*P* < 0.05). However, co-treatment of cells with both herbal formulations resulted in a significant decrease in LPS-induced IL-6 transcription in a concentration-dependent manner (*P* < 0.05). More specifically, expression of TNF-*α* in LPS-treated cells was inhibited by 54% and 77% by the lower and higher concentrations of FRAM-1, respectively, and by 77% and 95% by the lower and higher concentrations of FRAM-2, respectively. Notably, the level of IL-6 transcription in LPS + FRAM-2-treated cells at the higher concentration of FRAM-2 was significantly lower (*P* < 0.05) compared to all other treatment groups. 

### 3.4. Anti-Inflammatory Activities of the Fermented Herbal Formulations: Impact on Production of Key Inflammatory Cytokines

**Figure 8:**
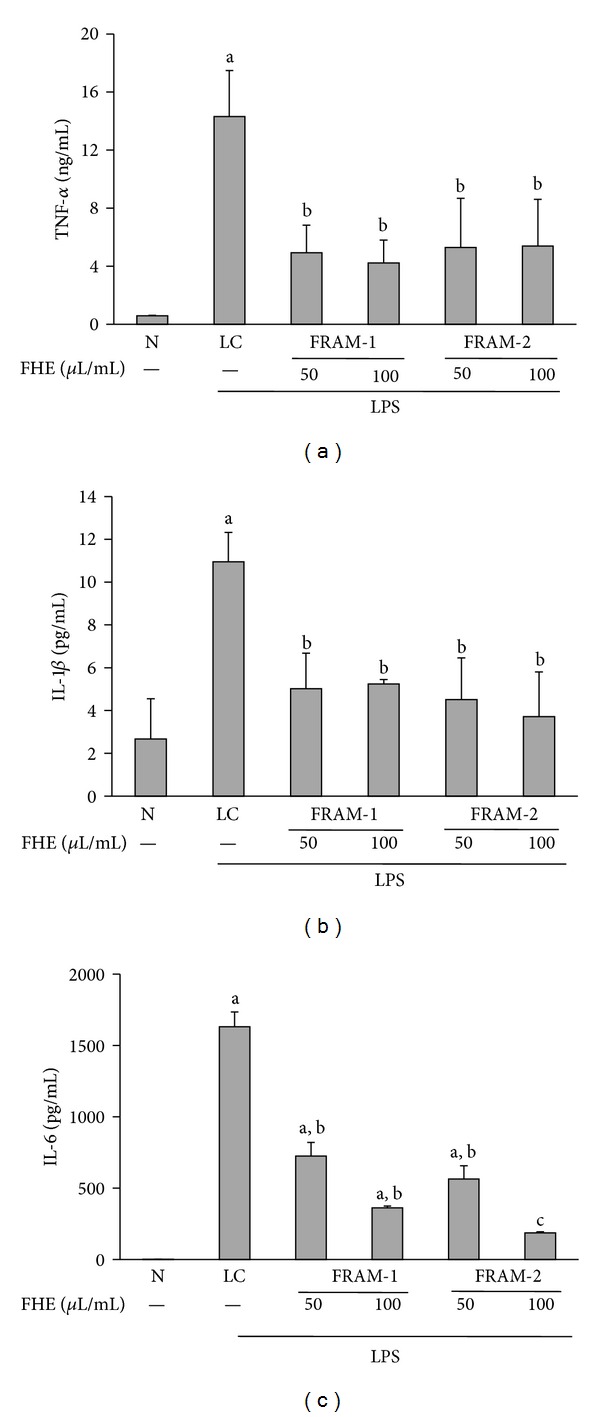
The effect of treatment with LPS in combination with saline (LC) or with two different concentrations of fermented Rhizoma Atractylodis Macrocephalae-1 (FRAM-1) and fermented Rhizoma Atractylodis Macrocephalae-2 (FRAM-2) formulations on production of TNF-*α* (a), IL-1*β* (b), and IL-6 (c) by RAW264.7 cells. The cells were treated for 24 h with the indicated concentrations of FRAMs. The control cells (N) and the noncontrol cells that were assigned to treatment with LPS alone (LC) were treated with sterile saline instead of herbal extracts. After this treatment, the noncontrol and control cells were treated with LPS (10 *μ*g/mL) and PBS, respectively, for 24 h; production of the above-mentioned cytokines was then determined. The detailed treatment regimen and experimental conditions are described in Section 2. The values are expressed as the mean ± SD, *n* = 3. The data for expression of the IL-6 gene were log-transformed prior to analysis by ANOVA. ^a^Statistically significant difference compared to control cells (*P* < 0.05); ^b^Statisticaly significant difference compared to cells treated with LPS plus saline (*P* < 0.05); ^c^significantly lower compared to all other treatment groups (*P* < 0.05). FHE, Fermented herbal extract.

Treatment of cells with LPS resulted in a marked increase in TNF-*α* production (24-fold, Figure 8(a)). However, co-treatment of cells with both fermented herbal formulations induced significant attenuation of LPS-induced production of TNF-*α*, accounting for 66% and 70% depletion by the lower and higher concentrations of FRAM-1, respectively, and 63% and 62% decrease by the lower and higher concentrations of FRAM-2, respectively. Accordingly, production of TNF-*α* did not vary significantly among or between the LPS + FRAM-1 and LPS + FRAM-2 treatments.

Treatment with LPS resulted in a substantial increase in the production of IL-1*β* in the cells (4.1-fold, Figure 8(b)). Notably, co-treatment of cells with both fermented herbal formulations induced a significant decrease in LPS-induced production of IL-1*β*, resulting in 54% and 52% attenuation by the lower and higher concentrations of FRAM-1, respectively, and depletion of 59% and 66% by the lower and higher concentrations of FRAM-2, respectively. Thus, production of IL-1*β* did not vary significantly among or between the LPS + FRAM-1 and LPS + FRAM-2 treatments. 

Treatment with LPS resulted in a remarkable increase in production of IL-6 (1745-fold, Figure 8(c)), the level of which was reduced by 56% and 78% by the lower and higher concentrations of FRAM-1, respectively, and attenuated by 65% and 89% by the lower and higher concentrations of FRAM-2, respectively. Notably, at the lower concentration of the fermented herbs, production of IL-6 did not differ significantly between the LPS + FRAM-1 and LPS + FRAM-2 treatments, while at the higher concentration of the fermented herbs, the level of IL-6 in the LPS + FRAM-2 treatment was significantly lower than that of the LPS + FRAM-1 treatment.

### 3.5. Inhibitory Effect of FRAM-2 on LPS-Induced NF-*κβ* Activity

**Figure 9:**
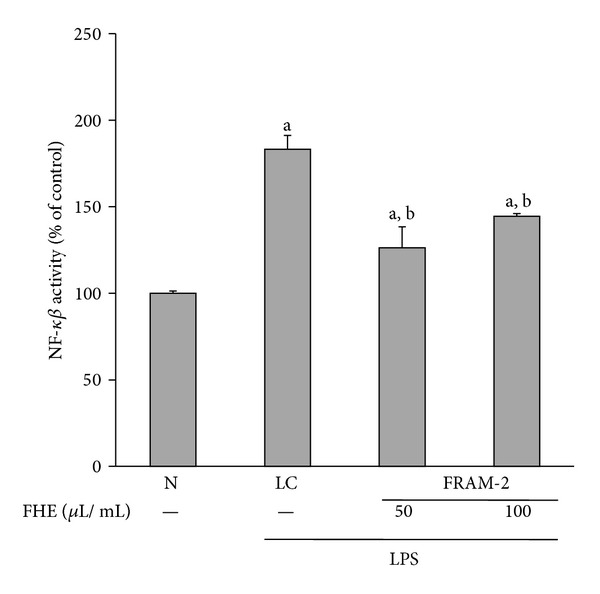
The effect of treatment with LPS in combination with saline (LC) or with two different concentrations of fermented Rhizoma Atractylodis Macrocephalae-2 (FRAM-2) on NF-*κβ* activity in RAW264.7 cells. The cells were treated with the indicated concentrations of FRAM-2 for 24 h. The control cells (N) and the noncontrol cells that were assigned to treatment with LPS alone (LC) were treated with sterile saline instead of herbal extract. After this treatment, the noncontrol and control cells were treated with LPS (10 *μ*g/mL) and PBS, respectively, for 1 h; the activation of NF-*κβ* was then determined. The detailed treatment regimen and experimental conditions are described in Section 2. The level of NF-*κβ* activity in the control cells (N) was set to 100%. The values are expressed as the mean ± SD, *n* = 3. The data were log-transformed prior to analysis by ANOVA. ^a^Statistically significant difference compared to control cells (*P* < 0.05); ^b^statistically significant difference compared to cells treated with LPS plus saline (*P* < 0.05). FHE: fermented herbal extract.

Because FRAM-2 produced stronger suppression than FRAM-1 on most of the LPS-induced inflammatory parameters, we selected FRAM-2 as the representative FRAM formulation for evaluation of the impact of fermented RAM on the NF-*κβ* activity of LPS-induced cells. As expected, treatment of cells with LPS resulted in an 84% increase in the activity of NF-*κβ*, as measured by the nuclear translocation of its p65 subunit (Figure 9). Notably, both the lower and higher concentrations of FRAM-2 induced significant inhibition of LPS-induced NF-*κβ* activity (31% and 21%, resp.). However, no significant difference in this effect was observed between the concentrations. 

## 4. Discussion

The intestinal epithelium, which is composed of a single layer of cells, plays several vital physiological roles. For example, it serves as a barrier to prevent passage of harmful intraluminal entities, including foreign antigens, microorganisms, and their toxins [1]. In addition, it also functions as a selective filter, permitting the translocation of essential dietary nutrients, electrolytes, and water from the intestinal lumen into the circulation [1]. However, the intestinal barrier function can be impaired in many diseases and due to exposure to a number of drugs and chemical agents, such as LPS [45]. Treatment with certain herbs, including RAM, has been found to protect the gut barrier in a number of diseases, including gastroenteritis, acute cholangitis, and MODS [25, 46, 47]. Exposure to RAM has been shown to prevent viral gastroenteritis through protection of intestinal mucosal cells against injury and improvement of the absorptive function [25]. 

We first evaluated the adverse effects of LPS on the intestinal barrier function *in vitro* using a monolayer of HCT-116 cells as a model and attempted to determine whether RAM and FRAM could protect IECs from the negative impact of LPS. As expected, LPS induced a marked increase in the permeability of cells, which was reflected in both TEER and HRP flux measurements. This is in keeping with the findings of an earlier *in vitro* study on human intestinal Caco2 cells [48], and *in vivo* experiments where LPS treatment resulted in intestinal barrier damage and augmentation of gut permeability [18, 19, 45], which in turn could trigger bacterial translocation from the gut lumen to mesenteric lymph nodes or other organs [4, 5]. In our study, coexposure of LPS-treated cells to RAM at the highest experimental concentration (200 *μ*L/mL) resulted in a definite, but insignificant, increase in TERR (39% enhancement) and a slight depletion of HRP-flux (7% decrease). In contrast, co-treatment with a similar concentration of either FRAM-1 or FRAM-2 resulted in a significant increase in TEER and depletion of HRP-flux in LPS-treated cells, indicating the beneficial impact of fermentation on RAM in protecting the membrane integrity and barrier function of IECs against LPS insult. This finding is in agreement with our previous *in vivo* experiments on rats, where it was found that a mixture of RAM and other herbs in fermented condition, but not in unfermented state, could significantly attenuate LPS-induced gut permeability [18]. Notably, the overall profile of barrier function and permeability assessment in the current study indicate that FRAM-2 is more potent than FRAM-1 in protecting IECs from endotoxic shock induced by LPS. This suggests that the conditions of fermentation could influence the protective role of FRAM against LPS insult.

It has been shown that LPS can directly interact with IECs via Toll-like receptor [8]. LPS is known to impose cellular oxidative stress [9] through the generation of ROS [10] and can promote barrier dysfunction via an oxidative mechanism [15]. Disruption of epithelial tight junctions (TJs) and induction of epithelial cell apoptosis have been reported to be the prime factors responsible for ROS-mediated damage to barrier integrity [11–13]. Accordingly, it is conceivable that antioxidants might play an important role in protecting the intestinal barrier against LPS insult. Substantial evidence has demonstrated the antioxidant activities of plants and herbs [49, 50], including RAM [23, 24].

To evaluate the antioxidant activity of RAM and the effect of fermentation on it, we measured the radical scavenging activity of RAM and FRAMs *in vitro*. Accordingly, treatment with RAM resulted in the scavenging of DPPH radical by approximately 23% at the prevailed sample concentration, which was increased significantly upon prior fermentation of the herbs, regardless of the condition of fermentation. Fermentation has been shown to augment the DPPH radical scavenging activity of many plants, vegetables, and plant products [27, 28]. Fan et al. [51] reported an association of a number of changes in macromolecular structure and composition, including an increase in the hydrophobicity of peptides and enhancement of the level of aromatic amino acids and histidine, cysteine, acidic, and/or basic amino acids with fermentation-mediated augmentation in antioxidant activities of soybean product. On the other hand, Đorđević et al. [28] reported that fermentation increases the phenolic content of plant products, and Kähkönen et al. [52] showed that plant polyphenolic compounds exert multiple biological effects, including antioxidant activity. This is in keeping with the study by Shan et al. [29], who reported a positive correlation between polyphenols and antioxidant activities of the herbs. These reports are also in agreement with our findings, where, in addition to enhancing the antioxidant activity, fermentation under either condition also resulted in significant elevation of the polyphenol content of RAM. The antioxidant activity of phenols has been shown to be attributed to their redox properties, which can play a crucial role in absorbing and neutralizing free radicals, quenching singlet and triplet oxygen, or decomposing peroxides [53].

It has been reported that LPS can cause a significant inflammatory response in IECs [54], which may also lead to increased epithelial permeability [20]. It is conceivable that free radicals and reactive oxygen metabolites produced during LPS insult can trigger and/or amplify inflammation via upregulation of expression of a number of genes, including NF-*κβ*, which, in turn, amplifies the inflammatory response by upregulating the production of several proinflammatory cytokines and enzymes, such as IL-1, IL-6, TNF- *α*, and iNOS [21, 22]. Polyphenols have been shown to enhance epithelial barrier functions [55] and exert anti-inflammatory effects in inflamed human intestinal epithelium [56]. Polyphenols can exert their anti-inflammatory properties at multiple levels via modulation of MAPK, Akt, and NF-*κ*B signaling pathways, suppression of production of inflammatory cytokines and chemokines, inhibiting the activity of COX and iNOS, and attenuating production of ROS/RNS [57]. Taking the above information into consideration, it is conceivable that because of their higher antioxidant activity and polyphenol content, fermented preparations of RAM may also protect IECs against LPS insult through the inhibition of inflammatory responses. This led us to conduct an in-depth study in order to assess the anti-inflammatory activities of FRAMs *in vitro* using the RAW264.7 cell line as a model. 

The inflammatory mediator NO plays a vital role in almost every stage of development of inflammation. NO also disrupts the intestinal barrier through a number of mechanisms, including direct epithelial injury via membrane peroxidation, induction of apoptosis in IECs via activation of the proapoptotic factor, procaspase 3, and damaging mitochondria, leading to the release of cytochrome C and DNA fragmentation [3]. In addition, NO reacts with superoxide (O_2_^−^) to produce the potent oxidant peroxynitrite (ONOO^−^), which leads to much of the cytopathic damage attributed to NO, including oxidation of sulfhydryls and peroxidation of membrane lipids [3]. Generation of NO is enzymatically catalyzed by inducible nitric oxide synthase (iNOS), whose expression is triggered by LPS treatment in many cell types, tissues, and organs, including gut [15]. In our study, exposure to LPS consistently provoked a marked increase in iNOS transcription and NO production in RAW264.7 cells, which were significantly inhibited when the cells were cotreated with either of the FRAMs at both experimental concentrations. The NO-suppressing effects of fermented food and other products of plants or herbs with anti-inflammatory properties have been well documented [18, 19, 30, 32, 33]. Notably, in the current study, among the FRAMs, FRAM-2 induced maximal inhibition of the above-mentioned LPS-induced parameters, in keeping with the stronger protective effect of FRAM-2 on IECs against LPS-insult.

The rate-limiting enzyme COX-2, which is involved in the synthesis of a number of biologically active inflammatory mediators, including PGE_2_, plays a crucial role in the development and promotion of inflammation. Similar to iNOS, the expression of COX-2 is induced by LPS treatment in many cell types, including IECs, which is accompanied by higher lipid peroxidation and abnormalities in membrane integrity [58]. In our study, treatment with LPS led to a dramatic increase in COX-2 transcription and significant augmentation of PGE_2_ production in RAW264.7 cells. However, cotreatment with both FRAMs resulted in significant attenuation of the two above-mentioned parameters in LPS-treated cells. The COX-2 suppressing effects of fermented plants and herbs have been well documented [30, 34, 59]. Notably, the gross profile of our results showed that FRAM-2 is more potent than FRAM-1 in suppressing LPS-induced expression of COX-2, in parallel with the effect of FRAM-2 on iNOS expression in LPS-treated cells. 

Exposure of RAW264.7 cells to LPS also resulted in a marked increase in both gene expression and production of TNF-*α*, IL-1, and IL-6, which are the cytokines playing an important role in the inflammatory process [60–62]. Promotion of intestinal mucosal injury and augmentation of intestinal epithelial permeability by TNF-*α* and IL-1*β* has been demonstrated [63, 64]. On the other hand, although the biological role of mucosal IL-6 has not been completely elucidated, there is evidence to suggest that IL-6 may play a role in the development of increased intestinal permeability during shock and critical illness [65, 66]. Cotreatment of LPS-treated cells with both FRAMs resulted in significant attenuation of both gene expression and production of the above-mentioned cytokines, mostly in a concentration-dependent manner. Prior fermentation of many herb and plant products has been shown to reinforce their inhibition of induced production of inflammatory mediators, such as TNF-*α*, IL-1*β*, and IL-6 [31, 67]. Overall, our data suggest that FRAM-2 is more potent than FRAM-1 in combating LPS-induced expression and production of the above-mentioned cytokines, in parallel with the effect of FRAM-2 on iNOS and COX-2. 

The results described above suggest that both FRAMs can effectively protect against LPS-induced insult. However, FRAM-2 preparation, where glucose was used to support the bacterial growth during fermentation, conferred stronger anti-inflammatory activities, and exerted more effective protection to the IECs compared to FRAM-1. One possible explanation for such differential activities between the two FRAMs is that the degree of fermentation of RAM might be higher in glucose than in LB broth. Some strains of *Bacillus*, including *B. licheniformis*, have been reported to produce *α*-amylase (an enzyme that hydrolyzes starch to glucose and acts as a fermentation stimulant) when bacteria are grown on media containing glucose or other monosaccharides as the sole source of carbon or energy [68, 69].

Because NF-*κβ* plays a central role in inflammation [21, 22], which is mediated through an increase in nuclear translocation of p65 protein and depletion of cytosolic I*κβ* [70], we evaluated the question of whether the anti-inflammatory impact of the fermented RAM on LPS-treated cells is driven through the inhibition of NF-*κβ* activation. For this study, because FRAM-2 induced stronger suppression than FRAM-1 on most LPS-induced inflammatory parameters, we selected FRAM-2 as the representative FRAM formulation. According to our findings, both the lower and higher concentrations of FRAM-2 induced significant inhibition of LPS-induced NF-*κβ* activity. Our results are in agreement with those of earlier *in vitro* and *in vivo* studies where suppression of iNOS and COX-2 gene expression by fermented herbs or plant products was found to be mediated by the inhibition of NF-*κβ* activation [30, 71]. 

## 5. Conclusion

Our results highlight that the fermented RAMs possess appreciable antioxidant and anti-inflammatory activities and protect IECs against LPS-mediated insult. However, these beneficial properties of FRAMs are greatly influenced by the condition of fermentation. Based on the present findings on molecular mode of action of FRAMs against LPS-insult, it is also conceivable that our study needs future investigations to understand the exact chemical changes in RAM mediated by fermentation which improve the pharmacological activities of this herb. Although we have shown that polyphenol might be a major contributing factor to the antioxidant activity of FRAMs, the possible involvement of other chemical substances in the radical scavenging activity of FRAMs should also be judged by further studies. Additionally, previous studies have shown that atractylenolide I and atractylenolide III are the two major compounds in RAM that contribute to its anti-inflammatory activities [72, 73]. Accordingly, it would be worthwhile to evaluate whether fermentation could make any modification of these compounds in RAM extract. If such modifications would occur, further studies should be performed to scrutinize whether the modified compounds have stronger ant-inflammatory activities compared to the unfermented ones. Additionally, identification and characterization of other possible anti-inflammatory compounds from FRAMs using powerful analytical techniques such as LC-NMR/MS may lead to the development of therapeutic agents for treating inflammatory diseases.
